# IGF2BP3 Regulates TMA7-mediated Autophagy and Cisplatin Resistance in Laryngeal Cancer via m6A RNA Methylation

**DOI:** 10.7150/ijbs.80921

**Published:** 2023-02-22

**Authors:** Like Yang, Bingrui Yan, Lingmei Qu, Jingyuan Ren, Qiuying Li, Jingting Wang, Xuan Kan, Ming Liu, Yakun Wang, Yanan Sun, Chao Wang, Peng Wang

**Affiliations:** 1 Department of Otorhinolaryngology, Head and Neck Surgery, The Second Affiliated Hospital, Harbin Medical University, Harbin 150086, China.; 2 Department of Otorhinolaryngology, Head and Neck Surgery, The Fifth Affiliated Hospital, Harbin Medical University, Daqing 163316, China.; 3 Department of Otorhinolaryngology oral maxillofacial Head and neck Surgery, Jilin Tumor Hospital, Changchun, 130012, China.

**Keywords:** LSCC, TMA7, UBA2, m^6^A RNA modification, cisplatin resistance, autophagy.

## Abstract

Translation machinery associated 7 homolog (TMA7) is closely related to proliferation-related diseases. However, the function and regulatory mechanism of TMA7 in laryngeal squamous cell carcinoma (LSCC) remain unclear. The present study aimed to investigate the effect of TMA7 on the occurrence and development of LSCC and to study the mechanism of TMA7. TMA7 is upregulated in LSCC tissues and associated with poor prognosis. After TMA7 downregulation, the autophagy level was increased, and the proliferation, migration, and invasion of LSCC cells were inhibited. The m6A methylated reader IGF2BP3 enhanced the stability of TMA7 and reduced the level of autophagy. TMA7 interacted directly with UBA2. Furthermore, the activation of the IGF2BP3-regulated TMA7-UBA2-PI3K pathway is the primary mechanism by which TMA7 inhibits autophagy and promotes the progression of LSCC. The current study revealed that IGF2BP3-mediated TMA7 m6A modification promotes LSCC progression and cisplatin-resistance through UBA2-PI3K pathway, providing new insights into the autophagy-related mechanism, potential biomarkers, and therapeutic targets for LSCC.

## Introduction

Laryngeal cancer is the main pathological type of laryngeal squamous cell carcinoma (LSCC) and LSCC is the second most prevalent carcinoma in the head and neck region 1, 2. In 2020, the number of new cases of laryngeal cancer accounted for >180,000, and the number of new deaths accounted for approximately 100,000 worldwide 3. Although therapeutic methods have been greatly improved in the past decade, the 5-year overall survival rate dropped from 66% to 63% 4. The recurrence and resistance to chemotherapy or radiotherapy are the main causes of death in advanced LSCC patients. Therefore, effective markers for early diagnosis and therapy are an urgent requirement.

N6-methyladenosine (m6A) modification plays a major role in spermatogenesis, tissue development, cell differentiation, reprogramming, stress responses, and RNA-protein interactions by affecting the translation efficiency, RNA stability, and splicing in biological processes 5-7. Several enzymes associated with m6A, such as methyltransferases, effector proteins, and demethylases, dynamically regulate the m6A RNA modification. Accumulating evidence suggested that m6A modifications play a major role in various human cancers. Chen et al. demonstrated that WTAP participation in m6A methylation has a vital role in the occurrence of hepatocellular carcinoma 8. For instance, Liu et al. identified that the novel YTHDF1-EIF3C axis is critical for ovarian cancer progression and serves as a target for developing therapeutics for cancer treatment 9. The current study suggested that RBM15-mediated m6A modification of *TMBIM6* mRNA enhanced its stability in LSCC in an IGF2BP3-dependent manner 10.

Autophagy is an intracellular evolutionarily conserved catabolic degradation process in which cytoplasmic macromolecules, aggregated proteins, damaged organelles, or pathogens are delivered to lysosomes and digested by lysosomal hydrolases to generate nucleotides, amino acids, fatty acids, sugars, and ATP, followed by recycling into the cytosol 11-13. Accumulating evidence indicated a vital role of autophagy in cancer development and chemotherapy resistance 14, 15.

PI3K signaling is associated with various cancers in previous studies 16, 17, and the PI3K/mTOR signaling pathway regulates autophagy in cancer cells 18, 19. Therefore, inhibition of PI3K signaling is considered a promising approach for treating these diseases and a principal target for drug development 20.

We screened TMA7 by multi-omics analysis and immunohistochemical techniques in the present study. TMA7 was overexpressed significantly in LSCC tissues compared to paracarcinoma tissues and correlated with prognosis. TMA7 is also known as CCDC72 or HSPC016, the aberrant functions of TMA7 are closely related to diseases, especially tumor progression 21, 22. However, the regulatory mechanisms of TMA7 during tumor progression remain unexplored. Herein, we revealed that TMA7 plays an oncogenic role in LSCC. Mechanically, the m6A modification of TMA7 is regulated by IGF2BP3, which further induces autophagy and cisplatin resistance by regulating UBA2 in LSCC cells. Taken together, these results suggested that targeting the IGF2BP3/TMA7/UBA2 axis may be a novel and promising therapeutic approach for LSCC.

## Results

### *TMA7* oncogene is upregulated in LSCC tissues

**Figure 1:**
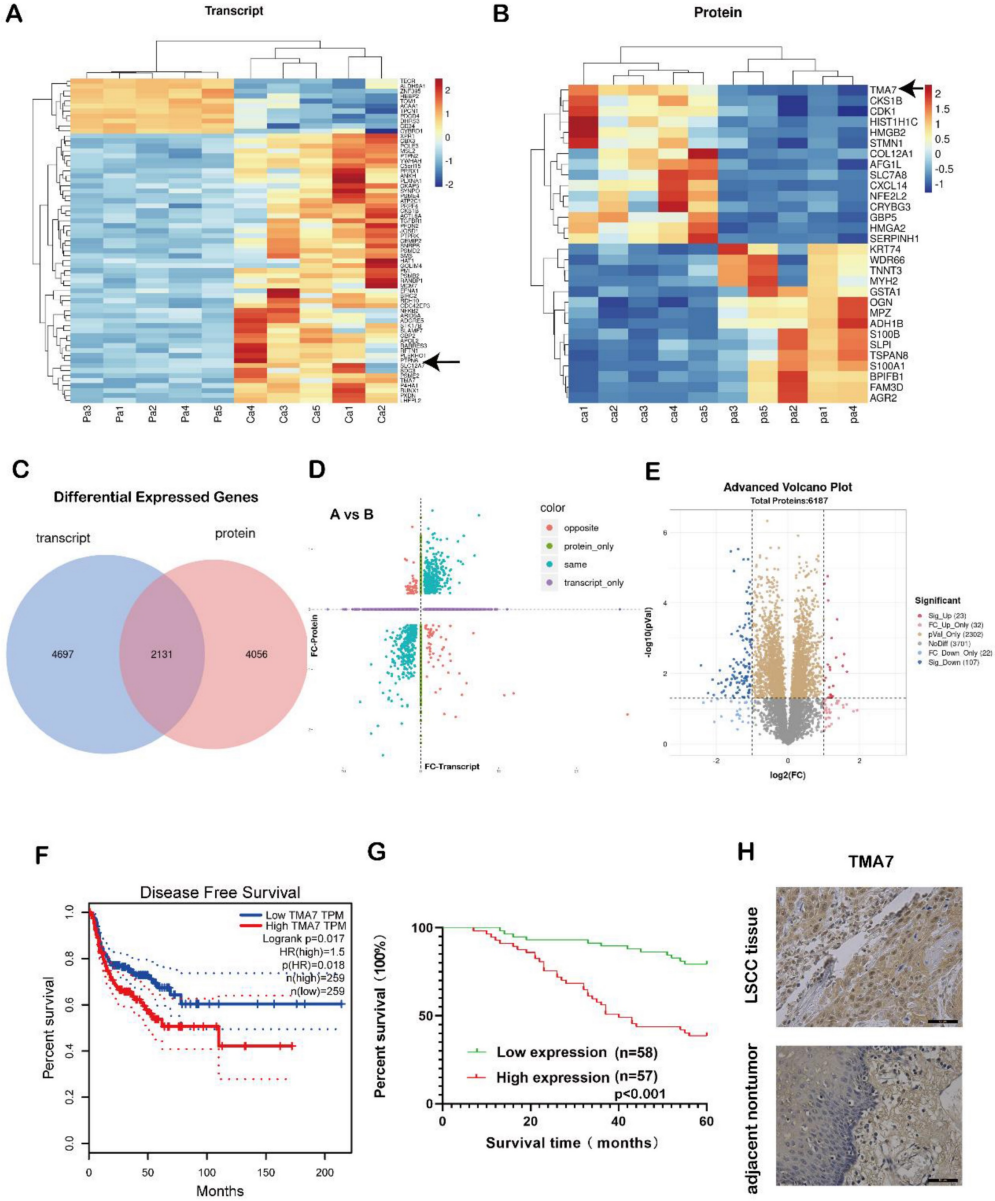
TMA7 as an oncogene in LSCC tissues is up-regulated. **(a)** Transcriptomic heatmap of differentially expressed genes. **(b)** Proteomic heatmap of differentially expressed proteins. **(c)** A collection of differentially expressed molecules co-transcriptomic and proteomic. **(d)** Transcriptomic and proteomic differential expression four-quadrant map. **(e)** Proteomic volcano plot of differentially expressed proteins. **(f)** Survival analysis levels of TMA7 predicted from GEPIA database. **(g)** Survival analysis levels of TMA7 predicted from 115 laryngeal cancer patients. **(h)** Representative images of TMA7 IHC results in LSCC tissue and para-carcinoma tissue, brown dots are TMA7 positive results. Scale bar, 20μm.

In order to study the gene expression, five pairs of LSCC and para-carcinoma tissue samples were selected for combined proteomic and transcriptomic analysis. We selected OmicStudio tools to analyze the data. The results are shown in Fig. 1a-e. In the transcriptomic analysis at log2FoldChange>1, or log2FoldChange<-1 (*p*<0.05), After removing extreme data, representative genes were elected for cluster analysis, 54 genes were upregulated significantly compared with paracarcinoma tissue, while 11 genes were down-regulated significantly (Fig. 1a, Supplementary additional file 1, transcript table). In the proteomic analysis results, at *p*<0.05, 2486 proteins exhibited significant differences. Under the same conditions, after removing extreme data, representative proteins were elected for cluster analysis, 15 proteins were upregulated significantly compared with paracarcinoma tissue, while 15 proteins were down-regulated significantly (Fig. 1b, Supplementary additional file 1, protein table).

The transcriptome analysis revealed that 6828 genes were differentially expressed in the patient samples compared to the adjacent tissues. A total of 6187 proteins were differentially expressed in the proteomic data. There were a total of 2131 genes that were differentially expressed in both sets of data (Fig. 1c). A correlation analysis of the two sets of data revealed differentially expressed genes in the transcriptomic and proteomic data, as shown in Fig. 1d. The blue dots represent two genes with the same omics expression trend. The volcano plot was also performed using the OmicStudio tools (Fig. 1e). In addition, we assessed the significance of the upregulated genes in LSCC tissue, the protein content in the tissue, and the differential expression fold under specific screening conditions and identified the genes with an upregulated expression (Fig. S1a-c).

Among these, the level of TMA7 was increased at both RNA and protein levels. Therefore, we selected TMA7 as the gene of interest. The statistical analysis of the data showed that in LSCC tissue, the level of TMA7 was higher than that in the paracarcinoma tissue at both the protein and RNA levels. The clinicopathological features were analyzed according to the expression of TMA7 in LSCC tissues (Table 1). As shown in Table 1, patients with high TMA7 expression of LSCC could achieve a high clinical stage (***p<0.001) and T classification (**p<0.01). In addition, patients with high TMA7 had lymph node metastasis (***p<0.001) and frequent recurrence (**p<0.01) (Supplementary additional file 2, Table S2). However, TMA7 expression was not significantly associated with age, sex, and primary location. Also, the Kaplan-Meier survival curves showed that increased TMA7 was correlated with poor prognosis 23 (Fig. 1f-g). Thus, IHC of TMA7 was conducted in LSCC and paracarcinoma tissues, as shown in Fig. 1h.

### TMA7 affects the proliferation, invasion, migration, and apoptosis of LSCC

**Figure 2:**
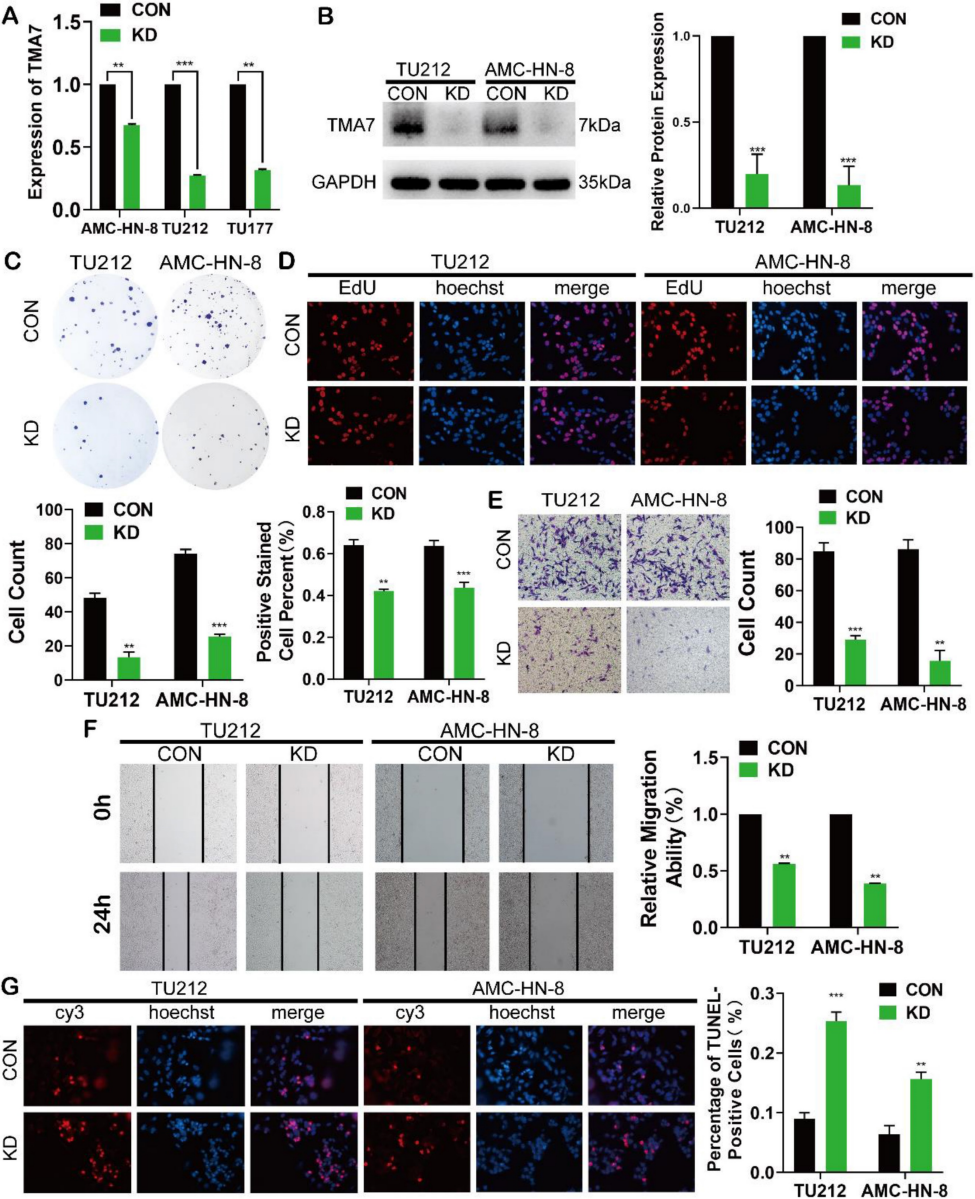
TMA7 affects the proliferation, invasion, migration and apoptosis of LSCC. **(a)** All the LSCC cells were transfected with lentivirus (shRNA targeting TMA7, also named as TMA7-KD), the expression of TMA7 of the stable cell lines was examined by RT-qPCR. **(b)** After cells transfecting, western blot was used to detect TMA7. **(c)** and** (d)** The proliferation ability of TMA7-KD LSCC cells was confirmed by colony formation assays (c) and EdU assays (d). **(e)** and** (f)** The abilities of invasion (e) and migration (f) of TMA7-KD LSCC cells were detected by transwell and wound healing assays. Scale bar, 200 μm. **(g)** Apoptosis was determined by TUNEL assay. Scale bar, 200 μm.

To elucidate the biological function of TMA7 in LSCC cells, we transfected the LSCC cell lines with lentivirus, termed as TU177, AMC-HN-8, and TU212, using shRNA to KD TMA7 expression (TMA7-KD). RT-qPCR and Western blot confirmed that TMA7 was successfully knocked down (Fig. 2a, b). Colony formation and EdU assays revealed that TMA7-KD inhibited cell proliferation (Fig. 2c, d). Transwell analysis showed that TMA7 KD reduced the invasiveness of LSCC cells (Fig. 2e). Subsequently, the migration ability of LSCC cells is decreased, followed by a decrease in TMA7 levels, as assessed by wound healing assay (Fig. 2f). After TMA7-KD in LSCC cells, the level of apoptosis was enhanced (Fig. 2g). In summary, these results showed that TMA7-KD weakened the proliferation, invasion, migration, and colony-forming ability of LSCC cells and increased the apoptosis.

### TMA7 is associated with autophagy of LSCC cells

To study the regulatory correlation between TMA7 and downstream molecules, we performed RNA-seq detection on three LSCC cell lines and their corresponding TMA7 KD cell lines. The expression levels of the relevant downstream RNA molecules changed. We also found that TMA7-KD upregulated 945 genes and downregulated 2521 genes at a cutoff of FC≥1.5 and *p*<0.05 (Additional file 3, Fig. S3a-d). Next, we screened out ubiquitin-like modifier activating enzyme 2 (UBA2), which effectuates the posttranslational modification of proteins by the addition of the small protein SUMO (see SUMO1; MIM 601912) or sumoylation that regulates protein structure and intracellular localization. We found that its mRNA level was reduced, and after GO analysis, it was related to protein binding. Database analysis revealed that it was expressed in the cytoplasm predicted from UniProt^24^. UBA2-KD promotes the apoptosis of clear cell renal cell carcinoma cells 25. Apoptosis-related phenotypes are discussed in this study, as apoptosis and autophagy are related to cancer 26. Therefore, we speculated whether UBA2 is related to autophagy in addition to apoptosis, which has not been described previously. In this study, the regulatory correlation between UBA2 and autophagy phenotype-related pathways was discussed, and *UBA2* was selected as a downstream gene of TMA7 for further research.

**Figure 3:**
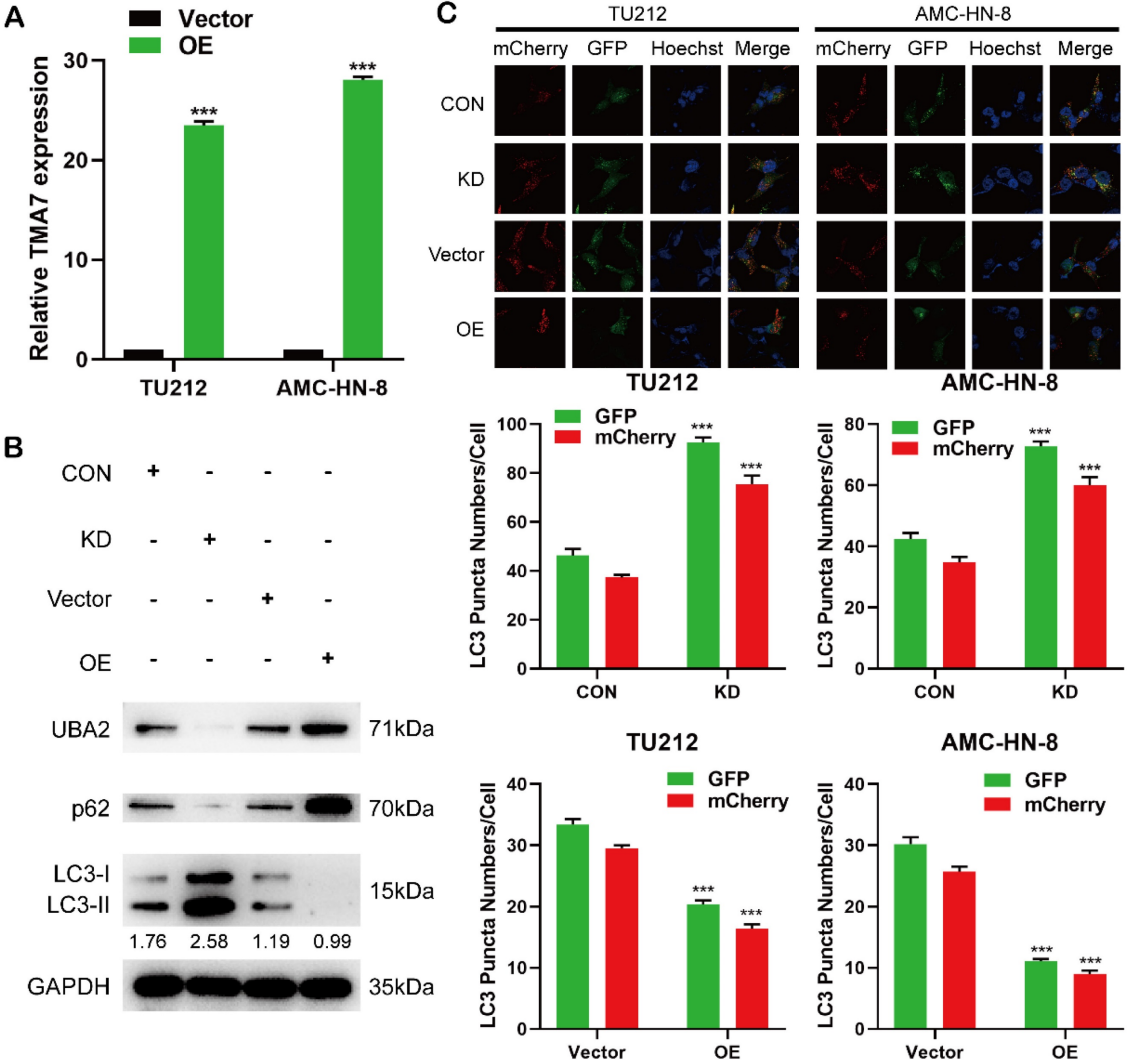
TMA7 is associated with autophagy in LSCC** (a)** LSCC cells were infected with TMA7-overexpressing lentivirus (TMA7-OE) or an empty control vector (Vector). RT-qPCR detection of TMA7 expression. **(b)** Western blot was used to detect UBA2, LC3B-II/I and p62 following TMA7 OE or KD. **(c)** After infected with TMA7 OE or KD lentiviruses, LSCC cells were labeled by mCherry-EGFP-LC3B and autophagic flux was analyzed by manual analysis.

In order to study the changes in the biological function in LSCC cells TMA7-OE, TMA7-OE lentivirus was transfected into the LSCC cells. RT-qPCR showed that TMA7 was correspondingly increased in LSCC cells after transfection with TMA7-OE lentivirus. (Fig. 3a). The lentiviruses of TMA7-OE were obtained from Genechem (MD, USA). In addition, we performed a Western blot to validate the OE and KD of TMA7 LSCC cells (Fig. 3b, Fig. S3e). The results showed that TMA7 was successfully overexpressed or silenced (Fig. 2b, Fig. 8a) in LSCC cells, and the protein content was increased and decreased correspondingly. To investigate autophagy and TMA7 in LSCC cells, we detected the autophagy-related proteins: p62, LC3B-I, and LC3B-II. After TMA7-OE, autophagy was attenuated, p62 expression was increased, and LC3B-II/I ratio was decreased. TMA7-KD provided contradictory results (Fig. 3b, Fig. S3e).

Autophagic flux was directly monitored using a tandem fluorescent LC3 reporter gene (mCherry-GFP-LC3) labeled with yellow autophagosomes (mCherry and GFP) and red autophagolyso- somes (mCherry). Therefore, yellow and red dots indicate autophagosome and autophagolyso- somes, respectively.

After infection with TMA7 OE or KD, LSCC and control cells were infected with adenovirus (mCherry-GFP-LC3, Hanheng, China). Confocal microscopy showed that the ratio of yellow to red puncta was constant in individual cells, while the number of dots increased. The analysis showed that the autophagy-lysosomal metabolism was not altered, and the overall level of autophagy was increased. Autophagy was increased after TMA7-KD and decreased after TMA7-OE (Fig. 3c). The expression of UBA2 decreased after TMA7-KD and increased after TMA7-OE (Fig. 3b). Therefore, we speculated that UBA2 may be a downstream molecule of TMA7 and was selected for further investigation.

### IGF2BP3 functions as an oncogene in LSCC cells

**Figure 4:**
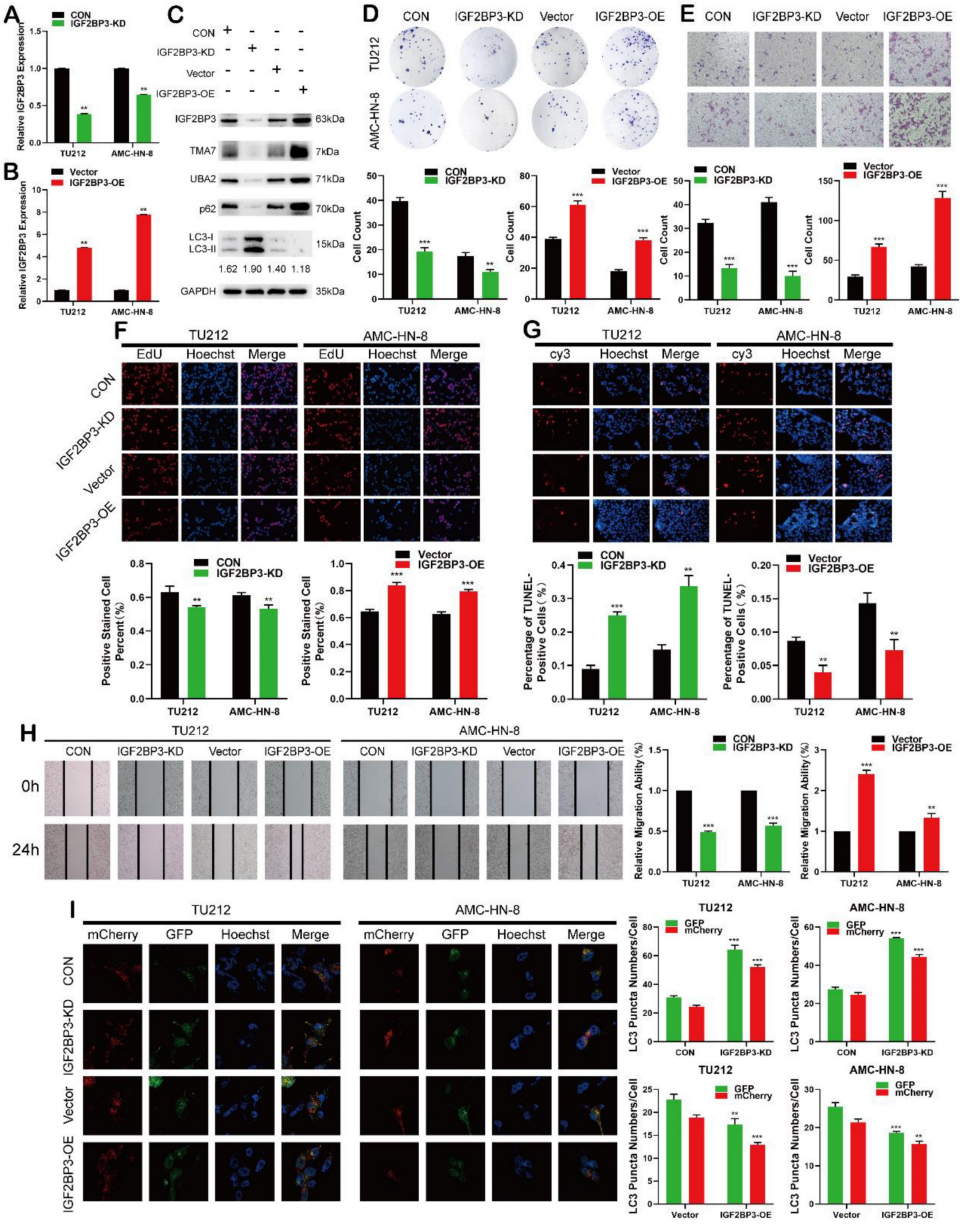
IGF2BP3 functions as an oncogene in LSCC cells. **(a)** and **(b)** Cells were infected with lentivirus overexpressing IGF2BP3 (IGF2BP3-OE) or transfected with shRNA targeting IGF2BP3 (IGF2BP3-KD). IGF2BP3 was detected by RT-qPCR. **(c)** Western blot was used to detect IGF2BP3, TMA7, UBA2, LC3B-II/I and p62 after IGF2BP3-OE or IGF2BP3-KD. **(d)** and **(f)** Colony formation**(d)** and EdU assays **(f)** were used to examine the proliferation of LSCC cells following IGF2BP3-KD or OE. **(e)** and **(h)** The invasive **(e)** and migratory **(h)** abilities of LSCC cells were observed by transwell and wound healing assay after IGF2BP3 OE or KD. Scale bar: 200 μm. **(g)** Apoptosis of cells was determined by TUNEL assay. I LSCC cells infected with IGF2BP3-OE or IGF2BP3-KD were labeled by mCherry-EGFP-LC3B. Autophagic flux was analyzed by manual analysis.

The analysis of differentially expressed genes from multiple datasets revealed that in addition to the upregulation of TMA7, IGF2BP3 was also upregulated in LSCC tissues. After IGF2BP3-KD or -OE in LSCC cells, the IGF2BP3 level changed successfully (Fig. 4a-b). Western blot showed that IGF2BP3, TMA7, and UBA2 decreased after IGF2BP3-KD, while the expression of IGF2BP3, TMA7, and UBA2 was increased after OE of IGF2BP3. To investigate the correlation between IGF2BP3 and autophagy in LSCC cells, we detected the autophagy-related proteins, p62 and LC3B. After IGF2BP3-OE, autophagy was attenuated, p62 was increased, and LC3B-II/I ratio was decreased. Conversely, the downregulation of IGF2BP3 resulted in opposite results (Fig. 4c, Fig. S4a).

Next, we studied the effect of IGF2BP3 on the proliferation, migration, invasion, and apoptosis on LSCC cells. Colony formation, EdU, wound healing, and Transwell assays showed that IGF2BP3-KD inhibited proliferation, migration, and invasion of LSCC cells (Fig. 4d-f, 4h), while IGF2BP3-OE promoted the opposite results. The apoptosis in LSCC cells was enhanced after IGF2BP3-KD and decreased after IGF2BP3-OE (Fig. 4g). The autophagy level was enhanced after IGF2BP3-KD and weakened after IGF2BP3-OE (Fig. 4I).

IGF2BP3 promotes LSCC cell migration, proliferation, and infiltration, reduces apoptosis and autophagy, and regulates the expression levels of TMA7 and UBA2.

### IGF2BP3 regulates TMA7 by m6A methylation

**Figure 5:**
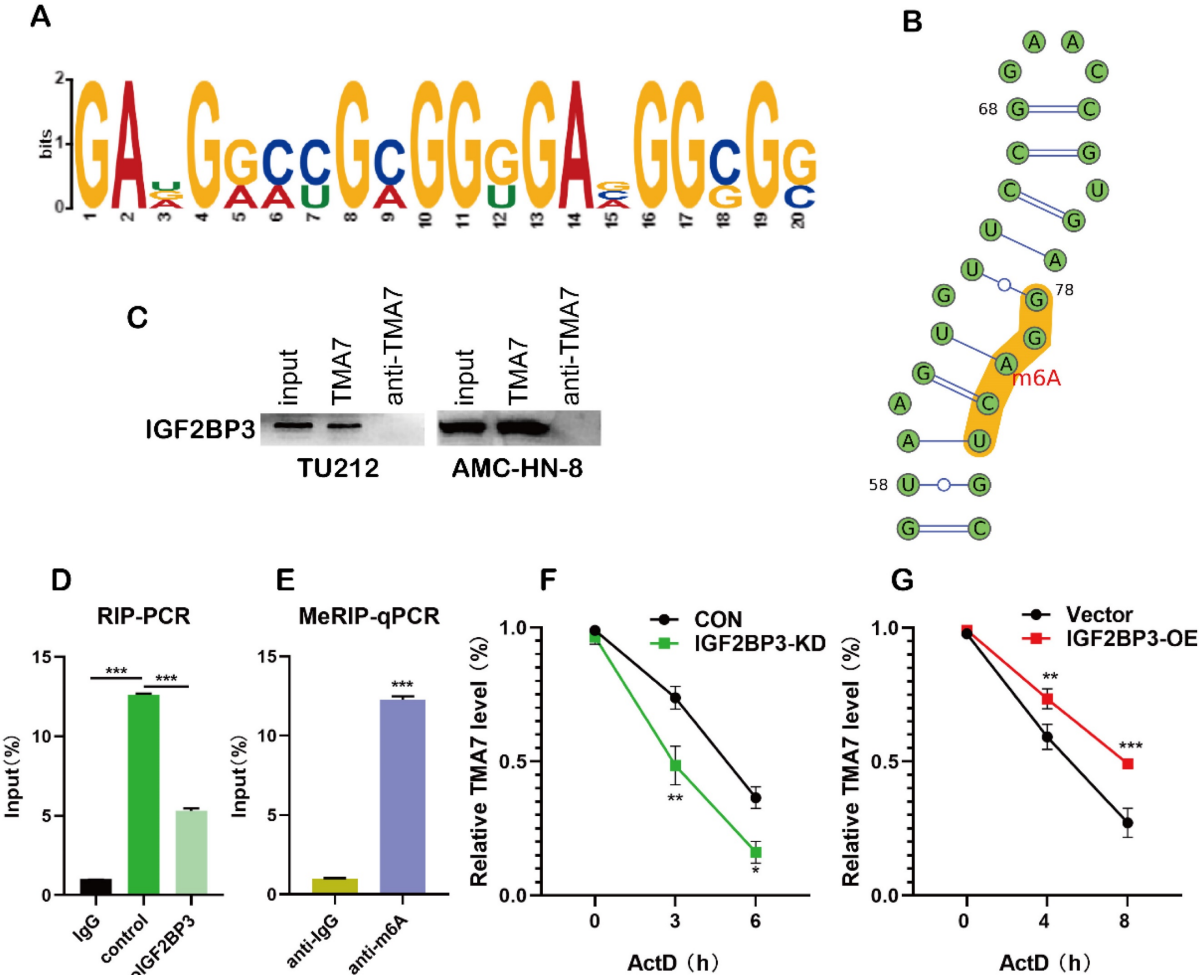
IGF2BP3 recognizes and binds TMA7 by m6A methylation**. (a)** and** (b)** The site where the TMA7 3'-UTR binds to IGF2BP3 corresponds to the m6A RNA modification element “RRACH” (R = A or G; H =A, U or C).** (c)** Binding of IGF2BP3 to TMA7 mRNA was detected by RNA pull-down (*p<0.05).** (d)** RIP detected binding of IGF2BP3 to TMA7 mRNA (**p<0.01)**. (e)** TMA7 mRNA was analyzed for m6A modification using MeRIP. (**p<0.01)**. (f)** and** (g)** RNA stability experiments showed that IGF2BP3 could regulate the half-life of TMA7 mRNA.

m6A plays a major role in mRNA regulation. The 3'-untranslated (UTR) sequence of *TMA7* mRNA contains a typical m6A modification motif, which is managed by bioinformatics analysis 27 (Fig. 5a, Fig. S5a-c). In order to predict the possible sites of m6A modification, we performed motif prediction on MEME 28 and obtained the m6A modification site sequence RRACH (D=A, G or U; R=A or G; H=A, U or C) 29 as predicted. The m6A methylation modification occurs on the A base in the motif (Fig. 5b). Western blot analysis indicated the existence of IGF2BP3 within the TMA7 sense RNA probe pull-down samples in TU212 cells (Fig. 5c). To prove the interaction between IGF2BP3 and TMA7, we performed RIP experiments, and the results showed that IGF2BP3 might bind to TMA7 (Fig. 5d). Next, we performed MeRIP in TU212 and identified an m6A modification site in the 3'-UTR region of TMA7. Therefore, we selected the above sequence and carried out the next step for verification. RT-qPCR analysis (*m6A* RIP) after MeRIP further confirmed the m6A-modified *TMA7* mRNA (Fig. 5e). Consistent with a potential role in promoting protein translation, reducing *TMA7* mRNA m6A modification decreases TMA7 protein expression and nascent TMA7 protein synthesis. (Fig. 4c). Subsequently, we concluded that IGF2BP3 directly binds to the mRNA molecule of TMA7. After OE or KD of IGF2BP3, Actinomycin D was used to detect the stability of *TMA7* mRNA on LSCC cells. Total RNA was extracted for RT-qPCR analysis as described previously. The mRNA expression at the indicated time points was calculated and normalized against that of *GAPDH*. Thus, we concluded that the ablation of IGF2BP3 can shorten the half-life of *TMA7* mRNA. IGF2BP3-OE prolongs the half-life of *TMA7* mRNA (Fig. 5f, g).

### UBA2 functions as an oncogene in LSCC cells

After analyzing the differentially expressed genes by RNA-seq (Fig. 3), we chose UBA2 as the downstream molecule of TMA7 for the next study after TMA7-KD.

**Figure 6:**
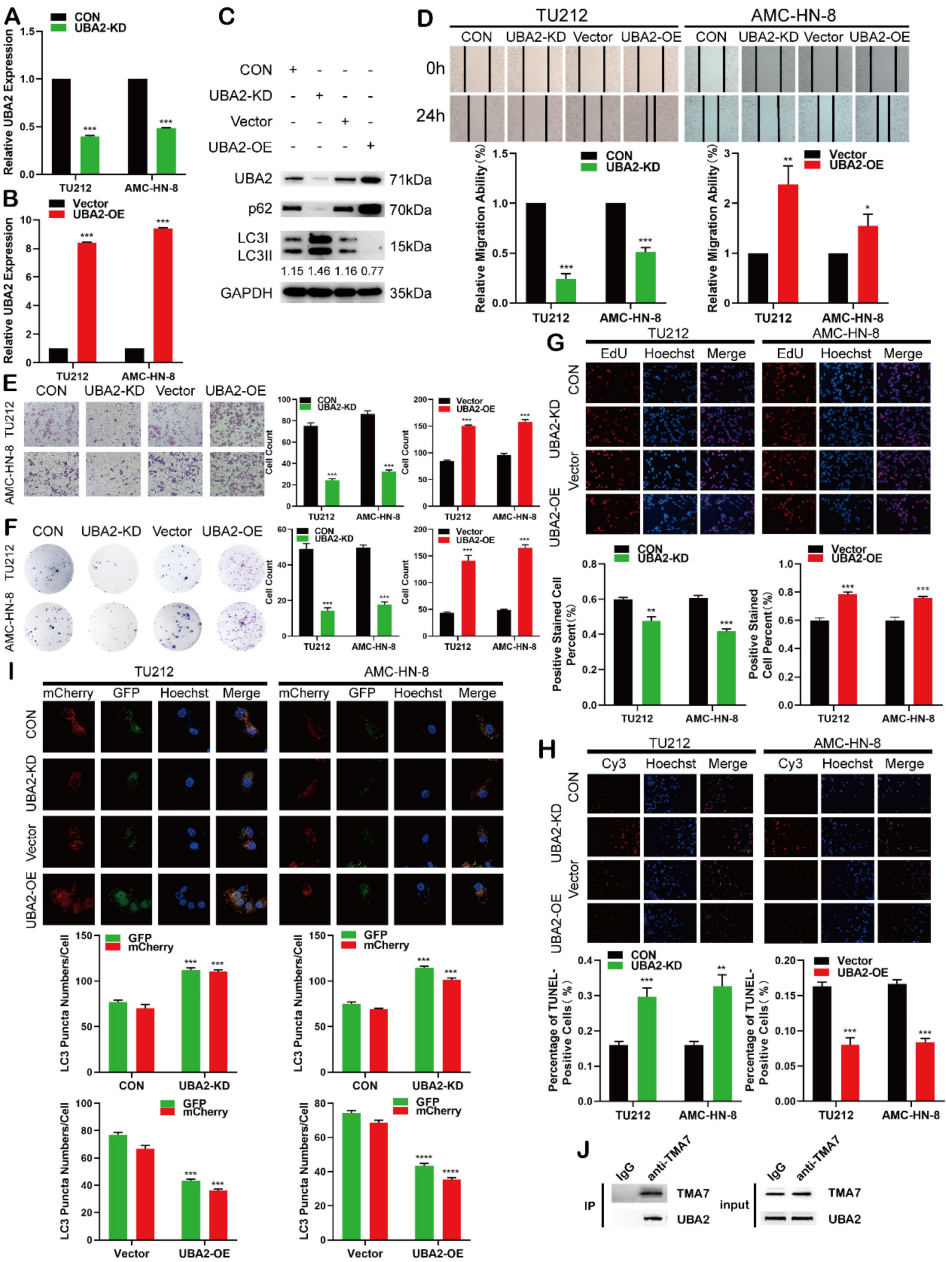
UBA2 functions as an oncogene in LSCC cells **(a)** and **(b)** RT-qPCR results of LSCC cells after UBA2 OE or KD **(c)** Western blotting results of UBA2, LC3B-II/I and p62 after UBA2 OE or KD. **(d)** and **(e)** The migratory (d) and invasive (e) abilities of UBA2 OE and KD on LSCC cells were evaluated by wound healing and Transwell assay. **(f)** and **(g)** Colony formation (f) and EdU(g) assays were used to examine the proliferation of LSCC cells after UBA2-KD or OE treatment. Scale bar, 200μm. **(h)** Apoptosis was detected by TUNEL. TUNEL-positive cells were scored by manual. Scale bar, 200μm. **(i)** LSCC cells after UBA2 OE or KD were labeled by mCherry-EGFP-LC3B adenovirus. Autophagic flux after UBA2 OE or KD was analyzed by manual. Scale bar, 25μm. **(j)** Co-immunoprecipitation (co-IP) of TMA7 with UBA2 in TU212.

Reportedly, UBA2 plays a major role in cancer 30-34, but its role in LSCC is yet unclear. The current data showed that UBA2-OE or UBA2-KD in LSCC cells altered UBA2 expression successfully (Fig. 6a-c). To elucidate UBA2 and autophagy in LSCC cells, we evaluated the autophagy-related proteins, p62 and LC3B. The OE of UBA2 decreased autophagy, increased p62, and decreased LC3B-II/I ratio. The disruption of UBA2 resulted in increased LC3B-II/I and decreased p62 (fig. 6c and Fig.S6a).

Next, we studied the effect of UBA2 on proliferation, migration, invasion, and apoptosis in LSCC cells. Colony formation, EdU, wound healing, and Transwell assays indicated that UBA2-KD inhibited proliferation, migration, and invasion in LSCC cells, whereas UBA2-OE promoted the difference (Fig. 6d-g). The apoptosis was enhanced after UBA2-KD and decreased after UBA2-OE (Fig. 6h). Furthermore, autophagy was increased when UBA2 was depleted, whereas autophagy was attenuated when UBA2 was overexpressed (Fig. 6I). A previous study proved that the level of TMA7 is associated with UBA2 (Fig. 3b); hence, we performed a co-IP experiment. The results showed that the two molecules interact directly (Fig. 6j), indicating that the two molecules may bind to each other. The results showed that *UBA2* acts as an oncogene in LSCC. A binding correlation was established between UBA2 and TMA7.

### TMA7 affects autophagy in LSCC through UBA2 and PI3K/mTOR pathway

**Figure 7:**
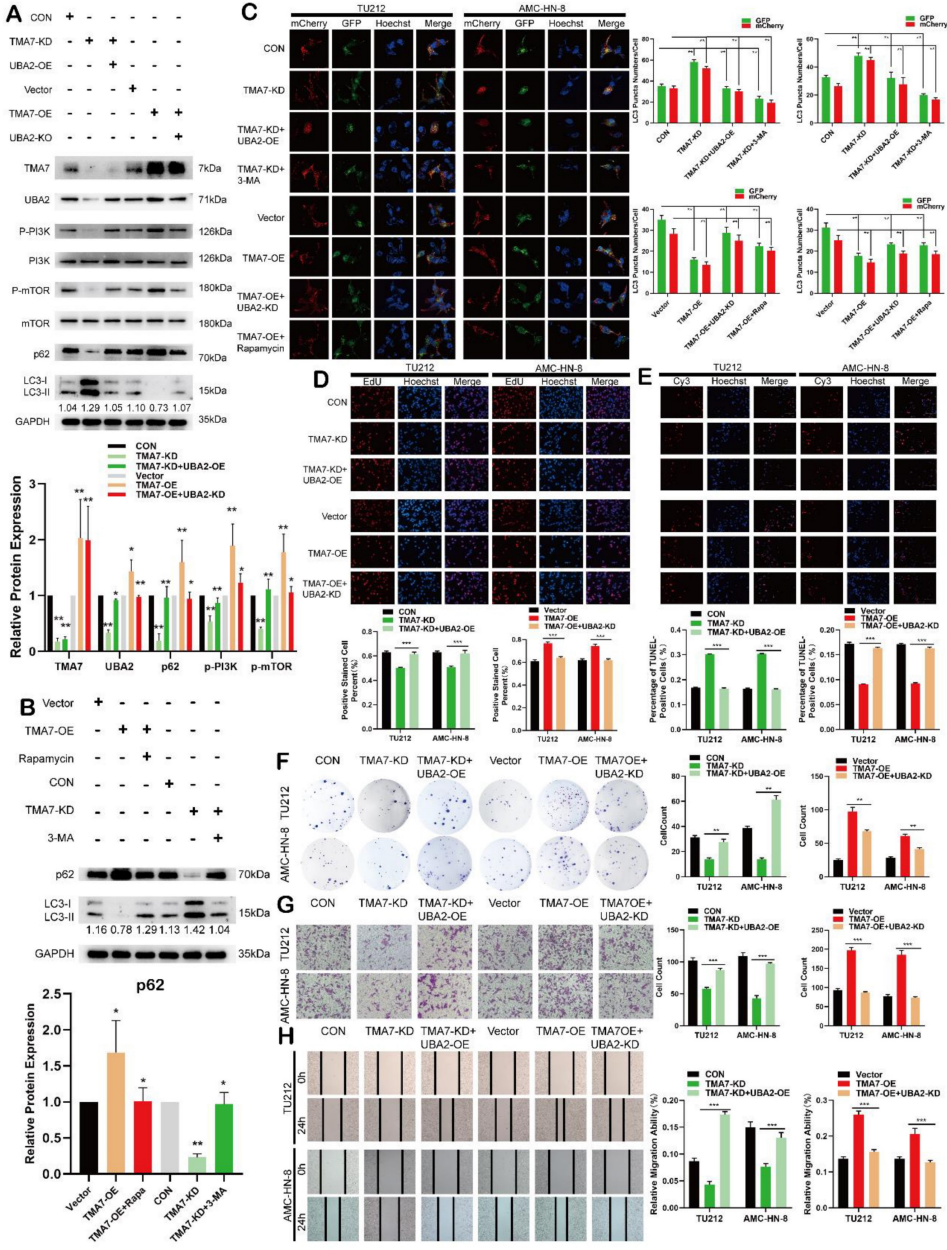
TMA7 affects autophagy in LSCC through UBA2 and PI3K/mTOR pathway **(a)** Western blot was used to detect TMA7 and UBA2 in TU212 transfected with TMA7-KD lentiviruses alone or in another way, transfected with TMA7-KD and infected with UBA2-OE. And another group was TMA7-OE, TMA7-OE with UBA2-KD. The levels of phosphorylated PI3K, phosphorylated mTOR, p62 and LC3B-I/II in TU212 were also determined by western blot. **(b)** Western blot was used to p62 and LC3B-II/I in TU212, transfection of TMA7-KD alone or TMA7-KD and 3-MA (2.5mM for 24h). And another group were divided into TMA7-OE, TMA7-OE and Rapamycin (100nM for 24h). **(c)** LSCC cells transfected with TMA7-KD alone or both TMA7-KD and UBA2-OE were labeled by mCherry-EGFP-LC3B for 48h, another group was TMA7-KD and 3-MA. LSCC cells transfected with TMA7-OE alone or both with TMA7-OE and UBA2-KD were treated under the same conditions, another group were transfected with TMA7-OE and Rapamycin. **(d)** and **(f)** LSCC cells were deal with TMA7-KD alone or with TMA7-KD and UBA2-OE lentiviruses, another group were Vector, TMA7-OE, TMA7-OE with UBA2-KD, cell proliferation was analyzed by colony formation (d) and EdU analysis (f). **(e)** The apoptosis of LSCC cells were detect by TUNEL assay. The cells were deal with TMA7-KD alone or with TMA7-KD and UBA2-OE, another group were TMA7-OE, TMA7-OE with UBA2-KD. Scale bar, 200μm. **(g)** and **(h)** Transwell (g) and wound healing assay (h) was used to determine the invasion and migration of LSCC cells, the groups were the same with colony formation assays. Scale bar, 200μm.

After TMA7-KD, we enriched the PI3K-mTOR pathway by RNA-seq and found that the PI3K-mTOR pathway is associated with the changes in TMA7. Therefore, we further studied the effect of TMA7 on the PI3K-mTOR signaling pathway. Western blot analysis showed that TMA7-KD decreased, whereas TMA7-OE increased the PI3K and mTOR phosphorylation levels (Fig. 7a). It is hypothesized that TMA7 can activate the PI3K-mTOR signaling pathway. Autophagy flux analysis showed that UBA2-OE attenuates the increase in autophagy caused by TMA7-KD, while UBA2-KD enhances the decrease in autophagy caused by TMA7-OE (Fig. 7c).

To assess the importance of the PI3K-mTOR signaling pathway for TMA7-mediated autophagy inhibition, we treated TMA7-OE LSCC cells with Rapamycin. Western blot and autophagy flux analysis indicated that inhibition of PI3K phosphorylation reactivates autophagy inhibited by TMA7-OE, and activation of PI3K phosphorylation re-inhibits autophagy activated by TMA7-KD when TMA7-KD LSCC cells are treated with 3-MA (Fig. 7b, c). Furthermore, UBA2-OE rescued the decreased cell proliferation, invasion, and migration, caused by TMA7-KD. Moreover, UBA2-KD rescued cell proliferation, invasion, and migration caused by TMA7-OE (Fig. 7d, 7f-h). UBA2-OE rescued the increased cell apoptosis caused by TMA7-KD, while UBA2-KD rescued the decrease in cell apoptosis caused by TMA7-OE (Fig. 7e). Therefore, we speculated that TMA7 interacts with UBA2, regulating LSCC autophagy levels through the PI3K/mTOR signaling pathway.

### IGF2BP3 regulates autophagy and UBA2 expression in LSCC through TMA7

**Figure 8:**
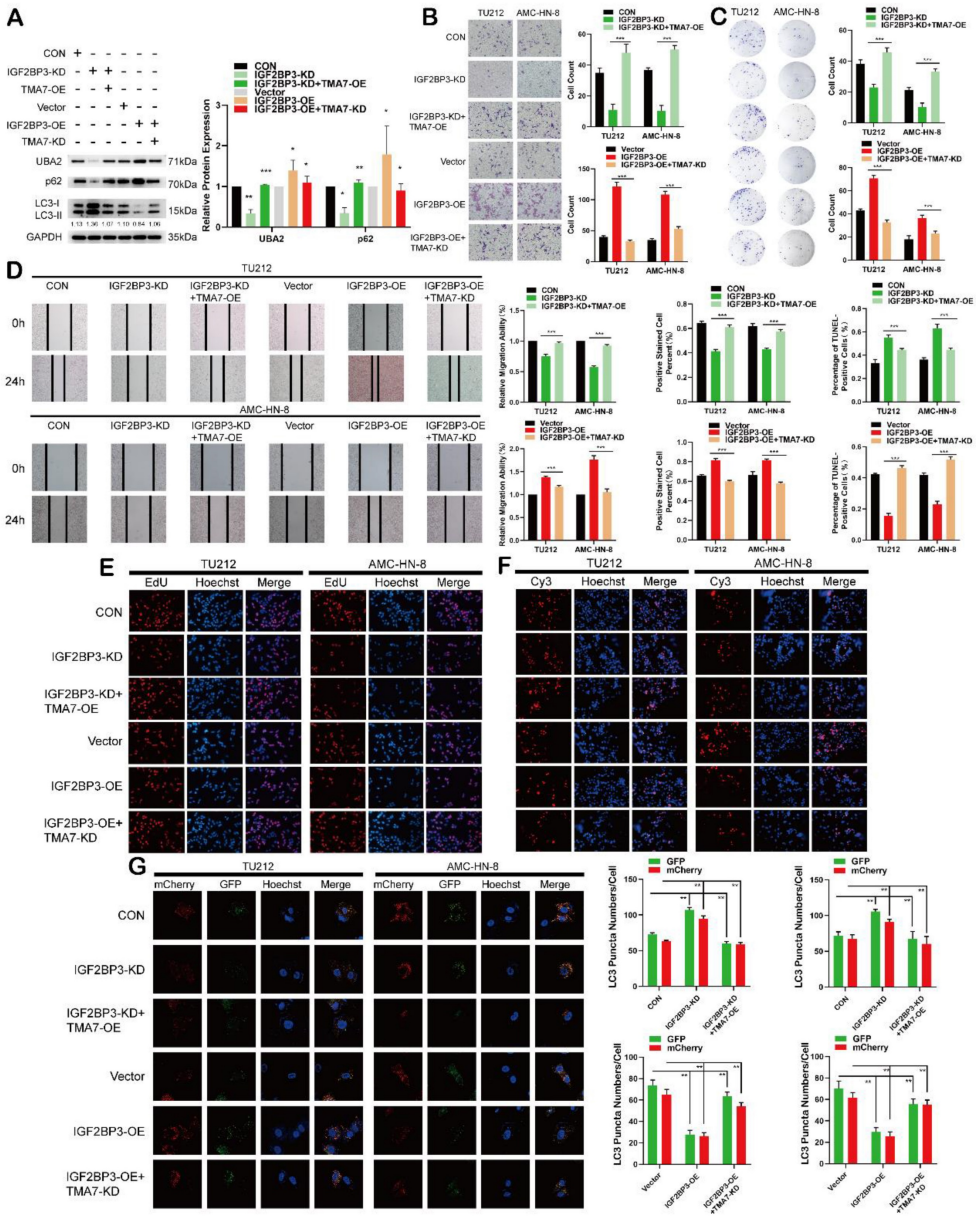
IGF2BP3 regulates autophagy and UBA2 expression in LSCC through TMA7 **(a)** The expression of IGF2BP3 and TMA7 in TU212 after IGF2BP3-KD alone or IGF2BP3-KD together with TMA7-OE were detected by western blot. UBA2, p62 and LC3B-II /I in TU212 cells were also detected in another group of TU212 with IGF2BP3-OE alone or with IGF2BP3-OE and TMA7-KD. **(b)** and **(d)** LSCC cells with IGF2BP3-KD alone or IGF2BP3 KD and TMA7 OE were detected by transwell (b) and wound healing assays (d) to study the abilities about invasion and migration, another group deal with IGF2BP3 OE alone or with IGF2BP3-OE and TMA7-KD. Scale bar, 200μm. **(c)** and** (e)** LSCC cells with IGF2BP3-KD alone or with IGF2BP3-KD and TMA7-OE. And another group of TU212 cells with IGF2BP3-OE alone or with IGF2BP3-OE and TMA7-KD. Proliferation of LSCC cells were studied by colony formation (c) and EdU analysis (e). **(f)** Apoptosis of LSCC cells were detect by TUNEL assay. The cells with IGF2BP3 KD alone or with IGF2BP3 KD and TMA7 OE, another group of cells with IGF2BP3 OE alone or with IGF2BP3 OE and TMA7 KD. Scale bar, 200μm. **(g)** LSCC cells with IGF2BP3 KD alone or IGF2BP3 KD and TMA7 OE were labeled by mCherry-EGFP-LC3B for 48h, autophagic flux was analyzed by confocal microscopy. Another group of LSCC cells with IGF2BP3 OE alone or IGF2BP3 OE and TMA7 KD were labeled by mCherry-EGFP-LC3B for 48h.

To verify the importance of TMA7 for IGF2BP3-mediated inhibition of autophagy, IGF2BP3-OE LSCC cells were infected with TMA7-KD and IGF2BP3-KD LSCC cells were treated with TMA7-OE lentivirus. Western blot showed that UBA2 upregulation and inhibition of autophagy due to IGF2BP3-OE were suppressed by TMA7-KD. While the downregulation of UBA2 and activation of autophagy from IGF2BP3-KD were rescued by TMA7-OE (Fig. 8a). Herein, TMA7-OE rescued the IGF2BP3-KD-induced decrease in cell proliferation, invasion, and migration. Conversely, TMA7-KD rescued the increase in cell proliferation, invasion, and migration induced by IGF2BP3-OE (Fig. 8b-e).

The results showed that TMA7-OE rescued the increase in cell apoptosis caused by IGF2BP3-KD. Also, TMA7-KD rescued the decrease in cell apoptosis caused by IGF2BP3-OE (Fig. 8f). The autophagic flux showed that TMA7-OE attenuated the increase in autophagy induced by IGF2BP3-KD. Conversely, TMA7-KD enhanced the decrease in autophagy induced by IGF2BP3-OE (Fig. 8g).

Therefore, we concluded an interaction between IGF2BP3 and TMA7, and IGF2BP3 regulates UBA2 expression and autophagy in LSCC via TMA7.

### TMA7 affects autophagy-induced cisplatin resistance in LSCC cells

**Figure 9:**
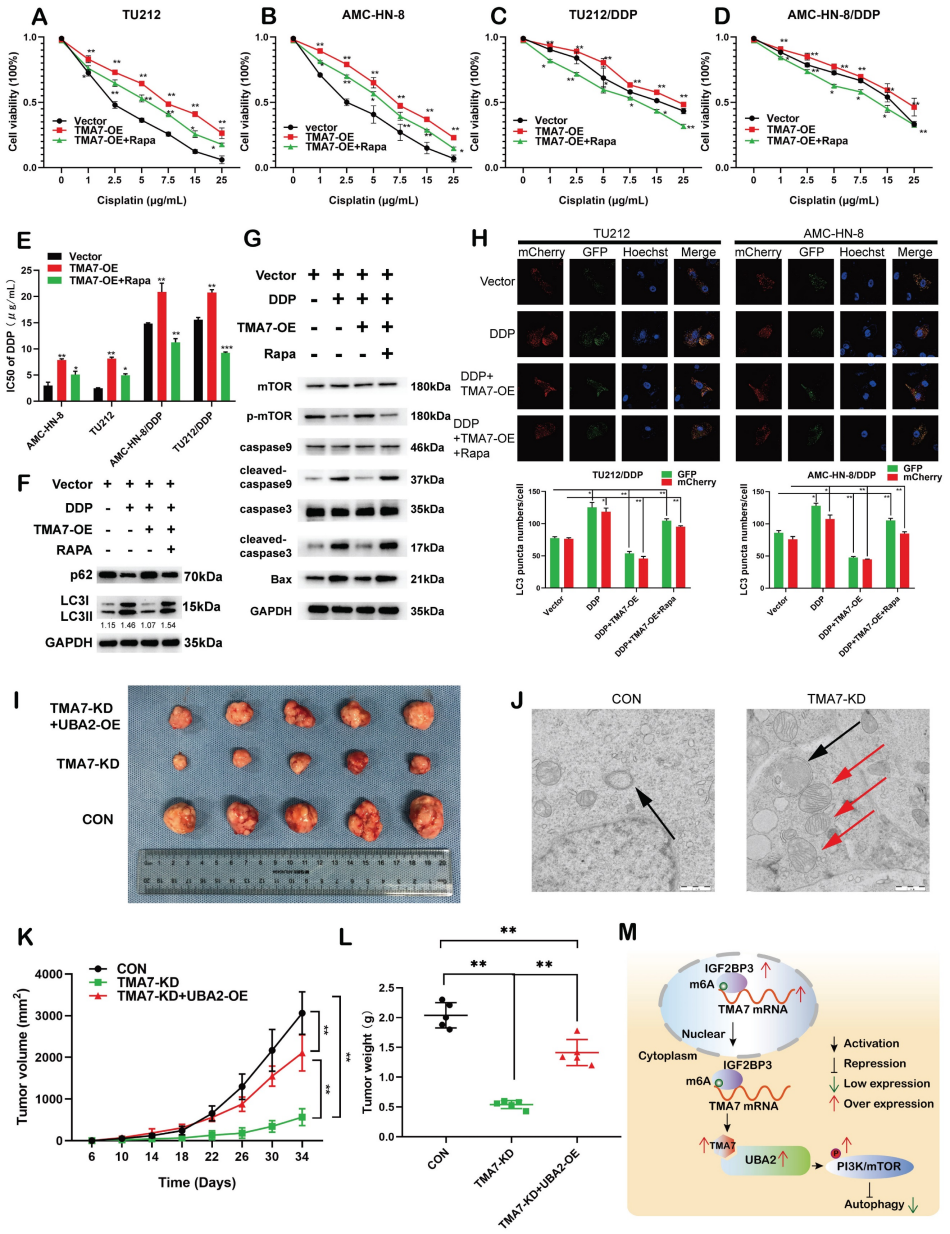
TMA7 affects autophagy-induced cisplatin resistance in LSCC cells **(a)** and** (b)** Relative viability of TMA7-OE or TMA7-OE+RAPA LSCC cells at indicated cisplatin concentrations for 24 hours. **(c)** and** (d)** Relative viability of AMC-HN-8/DDP and TU212/DDP cells after 24 h treatment with cisplatin. **(e)** The IC50 of TU212, AMC-HN-8, AMC-HN-8/DDP and TU212/DDP cells.** (f)** and** (g)** TMA7-OE TU212/DDP cells were treated with Rapamycin (100nM) for 24h, p-mTOR, cleaved-caspase9, cleaved-caspase3, BAX, LC3B and p62 were studied by western blot. **(h)** Cisplatin-resistant AMC-HN-8/DDP and TU212/DDP cells with TMA7-OE were labeled by mCherry-EGFP-LC3B for 48h. Another group of cells were then treated with Rapamycin. **(i), (k), (l)** Effects of TMA7 and UBA2 on tumor growth in LSCC xenografts *in vivo*. Representative images of tumors in nude mice following subcutaneous injection of TU212 cells containing TMA7-KD or cells containing TMA7-KD and UBA2-OE. Left: Tumor growth curve plotted from transplanted tumor volume data. Right panel: Tumor weight was measured after tumor resection.** (j)** Autophagosomes in LSCC cells were determined by TEM.** (m)** Schematic diagram of the mechanism by which TMA7 promotes malignant progression and drug resistance of LSCC by activating the PI3K-mTOR pathway and inhibiting autophagy. *P<0.05, **P<0.01.

To examine whether TMA7 modulates the chemosensitivity of laryngeal cancer to cisplatin, we constructed cisplatin-resistant AMC-HN-8/DDP and TU212/DDP cells. The results revealed that increased TMA7 promoted cell viability and induced chemoresistance in cells treated with cisplatin, whereas RAPA sensitized the cells to cisplatin treatment. Rapamycin might alter the effect of TMA7 on drug resistance in LSCC cells (Fig. 9a-d). RAPA decreased the IC50, while TMA7-OE increased the IC50 (Fig. 9e).

In addition, we replaced the autophagy inhibitor rapamycin, which inhibits autophagy and detects autophagy-related proteins. Western blotting revealed that TMA7-OE increased p62 and decreased the LC3B-II/I ratio, while rapamycin triggered the autophagy of cisplatin-resistant cells (Fig. 9f, Fig. S9a).

Next, we examined the effect of TMA7 on PI3K-mTOR signaling in cisplatin-resistant cells. Western blot indicated that TMA7-OE increased, while rapamycin decreased mTOR phosphorylation levels. Furthermore, TMA7-OE decreased the levels of BAX, cleaved-caspase3, and cleaved-caspase9, while rapamycin increased the levels of these proteins, indicating that due to the low level of apoptosis of cisplatin-resistant cells (Fig. 9g, Fig. S9g), TMA7-OE activated the PI3K-mTOR pathway in cisplatin-resistant cells.

Autophagic flux showed that rapamycin attenuated the decrease in autophagy induced by TMA7-OE (Fig. 9h). *In vivo*, nude mice were treated with LSCC cells transfected with TMA7-KD (n=5), another group of mice were treated with TU212 stable cells transfected with TMA7-KD and UBA2-OE (n=5), and the third group of mice was injected with TU212 cells. All nude mice were bred for 34 days. Xenograft data showed that low levels of TMA7 significantly inhibit xenograft growth and UBA2-OE enhances LSCC tumorigenicity (Fig. 9i, k, l).

Compared to the LSCC cells with no treatment, the autophagosomes in TMA7-KD cells viewed by TEM consisted of mitochondrial structures, indicating that TMA7-KD activates mitophagy and triggers subsequent autophagy (Fig. 9j).

## Discussion

LSCC is the most common head and neck malignancy, and advanced-stage patients have a poor prognosis 35. A comprehensive understanding of the molecular mechanism in LSCC is vital to finding new therapeutic strategies. In this study, multi-omics analysis of LSCC tissues was performed, and the results showed that TMA7 was significantly higher in LSCC tissues than in paracarcinoma tissues. A previous study showed increased TMA7 expression, which its KD inhibits the aggregation of dermal papilla cells 21, 22; however, there is no relevant study in LSCC. In the present study, we found that the overexpression of TMA7 is associated with poor prognosis in LSCC patients. Also, TMA7-KD significantly inhibited LSCC cell proliferation, migration, and invasion, suggesting an oncogenic role of TMA7 in LSCC. In addition, autophagy-related molecules, LC3II/I and p62, correlated with TMA7 expression levels, and TMA7-KD increased the overall level of autophagy in LSCC cell autophagy flux assays. The results showed that TMA7 is associated with the occurrence and development of LSCC and affects the autophagy level of LSCC via the expression of TMA7.

Autophagy is a cellular self-digestion program for aggregated protein degradation, and the removal of pathogen or impaired organelles by lysosomes is known as a type II programmed cell death 12. In addition, autophagy inhibits or promotes tumors in different states 36, 37. Gao et al. revealed that circPARD3-mediated regulation of autophagy promotes LSCC progression through the PRKCI-Akt-mTOR pathway 38.

Garcia-Mayea et al. found that the detection of autophagy-related proteins is valuable in assessing whether LSCC patients benefit from autophagy-suppression therapy 39. To explore the function of TMA7, we performed RNA-seq on LSCC cells with TMA7-KD and found that differentially expressed molecules were clustered in autophagy-related PI3K/mTOR pathway. In addition, we observed that the expression of *UBA2* gene was consistent with TMA7 in LSCC cells. UBA2 is a critical subcomponent of E1-activating enzymes and plays a major role in regulating SUMOylation in cellular processes *in vivo* 40. Interestingly, UBA2 plays a vital role in multiple diseases, such as colorectal cancer 30, breast cancer 32, and lung cancer 33. However, the role of UBA2 in LSCC tumor progression is unclear. The current study demonstrated that UBA2 downregulation inhibits LSCC cell proliferation and promotes autophagy and apoptosis, and the function of TMA7 on LSCC cells could be rescued and regulated by UBA2, suggesting that UBA2 is an oncogene of LSCC. The Co-IP results showed that TMA7 interacts with UBA2. Our results also revealed that TMA7 inhibits autophagy by activating the PI3K/mTOR pathway through UBA2 and ultimately increases the malignant potential of LSCC.

To elucidate the upstream regulatory mechanism, we further analyzed the multi-omics results from LSCC tissues and found significantly increased IGF2BP3 expression, which was consistent with TMA7 expression levels. IGF2BP3 is an m6A "reader" belonging to the IGF2BP family involved in the stability of methylated mRNAs 10. Recent studies have shown that IGF2BP3 exerts an oncogenic role via m6A modification in many cancers, such as colon cancer 41, bladder cancer 42, and breast cancer 43. In a previous study, we showed that in LSCC, IGF2BP3 expression is elevated and associated with poor prognosis 10. This study showed that IGF2BP3 downregulation promotes apoptosis and autophagy in LSCC. In addition, after IGF2BP3-KD, the levels of TMA7 and UBA2 were decreased, indicating that IGF2BP3 may regulate TMA7 and UBA2 expression. Furthermore, Western blot, PCR, and actinomycin D found that IGF2BP3 increased the stability of TMA7 through m6A modification. The rescue experiments showed that after IGF2BP3-KD, the attenuated oncogenicity of LSCC cells and the expression UBA2 could also be rescued by TMA7-OE, on the contrary, the expression of UBA2 upregulated by IGF2BP3-OE can be decreased by TMA7-KD. Taken together, we concluded that IGF2BP3/TMA7/UBA2 regulatory axis plays a pivotal role in LSCC malignant progression by modulating autophagy.

Accumulating evidence showed that autophagy activation is related to cancer chemoresistance and that inhibition of autophagy can significantly enhance the therapeutic efficacy 15. Cisplatin, also known as penicillin in cancer therapy, induces DNA damage and is the mainstay of chemotherapy for LSCC and other cancers 38, 44. Recent studies have shown that the development of cancer resistance decreases the efficacy of cisplatin and exhibits poor patient outcomes 45, 46. Thus, the chemotherapy resistance mechanism needs to be elucidated, and innovative therapeutic strategies are an urgent requirement. Godse et al. found that TMEM16A-OE inhibits the apoptotic activation in head and neck SCC, leading to cisplatin resistance 47. Zhou et al. demonstrated that downregulation of O-GlcNAc transferase enhances cisplatin-induced autophagy and promotes cisplatin-resistant ovarian cancer 48. However, the autophagy-related regulatory mechanism of cisplatin resistance in LSCC is yet unclear. In this study, we established a cisplatin-resistant LSCC cell line, tested autophagic flux, and observed increased autophagy in cisplatin-resistant cells. Also, TMA7-OE significantly enhanced the proliferation and inhibited apoptosis but Enhanced resistance to cisplatin in laryngeal cancer cells. These results suggested that TMA7 downregulation may be an effective strategy to improve the efficacy of cisplatin in the treatment of LSCC. Furthermore, electron microscopy revealed that LSCC cells developed mitophagy after TMA7 depletion at the subcellular level, suggesting that the current study might be based on the results of mitophagy. However, due to the limitation of research conditions, we will focus on this issue in successive studies.

## Conclusion

Taken together, the current study suggested that TMA7 is elevated in LSCC and is associated with a poor prognosis. TMA7 is an oncogene that regulates the proliferation, invasion, apoptosis, autophagy, and chemosensitivity of LSCC cells. TMA7 regulates the PI3K/mTOR pathway through UBA2, while IGF2BP3 regulates it in an m6a-dependent manner. Thus, this study might provide potential molecular markers and therapeutic targets for LSCC (Fig. 9m).

## Methods

### Tissue specimens

A total of 115 pairs of LSCC cancer tissues and para-carcinoma tissues were collected from LSCC patients who underwent partial laryngectomy or total laryngectomy in the Department of Otolaryngology, Second Hospital of Harbin Medical University (Harbin, China), from December 2015 to May 2017. All patients were first diagnosed with LSCC and then received total or partial laryngectomy with or without cervical lymph node dissection. No preoperative chemotherapy or radiotherapy was performed. LSCC tissues and para-carcinoma tissues from 115 patients were selected for IHC detection. Five additional matched tissues were detected by transcriptome microarray and proteomics. This study was approved by the Ethics Committee of Harbin Medical University (KY2017-047).

### Transcriptomics and proteomics multi-omics analysis

RNA-seq transcriptome sequencing and quantitative tandem mass tagging (TMT) proteomics were used to detect the five gene pairs and differences in protein expression from cancer tissues and adjacent LSCCs. RNA-sequencing based on the Illumina sequencing platform and proteomics services using Thermo Mass Spectrometry platform were carried out by Genechem (Shanghai, China).

### Immunohistochemistry

The tissue was embedded in paraffin, sliced into 4-μm-thick slices, and dewaxed after drying at 37 °C, followed by antigen retrieval. Protein was blocked with goat serum and incubated with primary antibody (TMA7 1:200; 20393-1-AP, Proteintech, Wuhan, China,) at 4 °C overnight, and biotinylated secondary antibody (goat anti-rabbit) at 24 °C for 1 h. Finally, the reactions were developed with DAB, hematoxylin counterstaining, dehydrated, mounted.

### Cell culture and cell transfection

TU212, TU177, and AMC-HN-8 were maintained in Dulbecco's modified Eagle's medium (DMEM) supplemented with 10% fetal bovine serum (FBS), 100 U/mL penicillin, and 100 mg/mL streptomycin and grown at 37 °C and 5% CO_2_. IGF2BP3, TMA7, and UBA2 control vectors, their knockdown (KD), and overexpression (OE) viruses were provided by Genechem. The transfection preformed according to the manufacturer's instructions. Subsequently, the cells were screened in a medium with 4 μg/mL puromycin for two generations to establish a stable transfected strain. The selection was continued under 2.0 μg/mL puromycin.

### Construction of cisplatin-induced drug-resistant cell lines

Cisplatin-resistant TU212 and AMC-HN-8 cells were obtained by serial exposure to cisplatin-containing medium. The concentration of cisplatin at which cells can survive is changed from 0.1 μg/mL to 4 μg/mL in four months. The cell lines were named TU212/DDP and AMC-HN-8/DDP, respectively.

### Real time quantitative polymerase chain reaction (RT-qPCR)

Total RNA was extracted from LSCC cells using TRIzol (Invitrogen, USA) and reverse transcribed using PrimeScript RT kit (TaKaRa, Dalian, China). Then, RT-qPCR was conducted in three replicates using FastStart Universal SYBR Green Master (ROX) (Roche) with *GAPDH* as an internal control. The relative expression of the target was calculated using the 2^-ΔΔCt^ method. The primers used for RT-qPCR are listed in Supplementary Table 2.

### Cell counting kit-8 (CCK-8) assay for cell viability and calculation of drug resistance index

An equivalent of 1000 cells/100 μL/well was grown in 96-well plates in a complete medium. Then, the medium was replaced by cisplatin-containing complete medium. After 24 h, the medium was discarded, the cells were gently washed with phosphate-buffered saline (PBS), and incubated with 100 μL of CCK-8 medium (10% of the total volume) at 37 °C for 2 h before measuring the absorbance at 450 nm.

The optical density (OD) values ​​of each well and the negative control well were compared, and the effects of various concentrations of cisplatin on TU212 and TU212/DDP cells were evaluated. The cell inhibition rate (IR) was calculated as follows: IR= [1-(absorbance of experimental wells-absorbance of blank wells)/ (absorbance of control wells-absorbance of blank wells)] ×100%. The drug concentration of cisplatin was used as the horizontal coordinate. The growth inhibition curves of TU212 and TU212/DDP cells were plotted using GraphPad Prism8 software, and the IC50 of cisplatin on different groups of cells was calculated. AMC-HN-8 cells were analyzed similarly.

### Colony formation assay

An equivalent of 200 cells/well was seeded in 6-well plates, and the medium was changed every 2 days. After 2 weeks, the surviving cells were fixed with 4% paraformaldehyde at 24 ℃ for 1 h and stained with 0.1% crystal violet.

### Transwell invasion assay

Transwell chambers (8.0-μm, Corning, NY, USA) were used for Transwell assays. Cells were suspended in 200 μL medium without FBS and seeded in the upper chamber precoated with 50 µL Matrigel Matrix (BD Biosciences). Then, the chambers were placed into 24-well plates with a 600 mL medium containing 20% FBS/well. After a 48-h incubation, the cells were fixed with 4% paraformaldehyde, and the chambers were stained with 0.1% crystal violet.

### Wound healing assay

A p200 pipet tip was used to create a scratch in the monolayer after the cells reached 95% confluency. The media was replaced with serum-free media for the next 24 h. The images were captured using digital microscopy (×20 magnification) at 0 h and 24 h. The migration ability was evaluated by measuring the scratch area without the cells.

### EdU assay

BeyoClick™ EdU Cell Proliferation Kit (Alexa Fluor 555) was utilized according to the manufacturer's instructions. An equivalent of 5000 cells/well was seeded in a plate of 96-well. EdU working solution was added to the wells on the following day, incubated for 2 h, and fixed with 4% paraformaldehyde for 0.5 h. After 5 min of permeabilization, the cells were treated with 50 μL EdU-Click reaction mix at 24 °C for 30 min. The cell nuclei were counterstained with Hoechst33342 (1 mg/mL; Beyotime Institute of Biotechnology, Jiangsu, China), and the images were captured under a fluorescence microscope (Nikon).

### Transferase-mediated dUTP nick end labeling (TUNEL) assay

TUNEL assay was carried out using the One Step TUNEL Apoptosis Assay Kit. An equivalent of 1×10^5^ cells/well were seeded in a plate of 24-well, fixed with paraformaldehyde (4%) for 30 min on the following day, permeabilized for 5 min, and incubated with the TUNEL detection reagent at 37 °C for 1 h in the dark. The cell nuclei were counterstained with Hoechst33342, and examined under a fluorescence microscope.

### Autophagic flux detection in cells

Tandem HBAD-mCherry-EGFP-LC3 was used to monitor the autophagic flux in cells as described previously 49. The cells were transduced with HBAD-mCherry-EGFP-LC3 adenovirus for 2 h, then the medium was refreshed with DMEM containing 10% FBS. Cultured for 48 h, and fixed with 4% paraformaldehyde for 30 min, followed by counterstaining with Hoechst33342. Confocal microscopy was used to demonstrate the autophagic flux in HBAD-mCherry-EGFP-LC3-expressing cells. The number of green and red puncta/cell was quantified (Image size: 101.41 μm×101.41 μm).

### Western blot

The Western blot was performed, as described previously 10. The primary antibodies were as follows: IGF2BP3 (1:1000; Proteintech, 14642-1-AP), TMA7 (1:1000; Proteintech, 20393-1-AP), UBA2 (1:1000; SANTA, sc-376305, USA), P62 (1:50000; Abcam, ab109012, USA), LC3B (1:2000; Abcam, ab192890), GAPDH (1:10^5^; Proteintech, 60004-1-Ig), mTOR (1:10000: Abcam, ab134903), p-mTOR (1:10000, Abcam, ab109268), PI3K (1:1000, Abcam, ab86714),p-PI3K (1:1000, Abcam, ab182651), caspase3 (1:5000, Abcam, ab32351), cleaved-caspase3 (1:500, Abcam, ab2302), caspase9 (1:10000, Abcam, ab32539), cleaved-caspase9 (0.5 µg/mL, Abcam, ab2324), and BAX (1:10000; Abcam, ab32503). The enhanced chemiluminescence kit (ECL, Beyotime, China) was used to visualize the signals. All signals were detected on a Tanon-5200 Imaging system. Image J (National Institutes of Health, Bethesda, MD) was used to integrate the relative densities of individual bands.

### RNA stability assays

LSCC cells were treated with Actinomycin D for 0, 3, 6 h or 0, 4, 8 h, and the cell lysates were subjected to RNA extraction. The subsequent RT-qPCR was carried out according to the standard protocols, and the target gene expression was normalized to that of *GAPDH*.

### RNA immunoprecipitation (RIP) assay

Magna RIP™ RNA-Binding Protein Immunoprecipitation Kit (Millipore, USA) was used to perform the RIP assay. The cells were lysed in RIP lysis buffer. The mixture was incubated with protein A/G magnetic beads conjugated with rabbit immunoglobulin G (17-700, Millipore), and IGF2BP3 antibody (1:100; Abcam, ab177477) was incubated at 4 °C overnight with cytolytic lysates supplemented with RNase inhibitor. After six washes, RNA was collected by the phenol-chloroform method. Finally, the RNA was detected by RT-qPCR.

### Methylated RNA immunoprecipitation-PCR (MeRIP-qPCR)

MeRIP-qPCR was performed according to our previous study 10. TMA7-specific primers were designed using the m6A site predicted site SRAMP 27.

### RNA pulldown assays

The RNA-protein pulldown kit (Pierce, USA) was used for RNA pulldown assays according to the manufacturer's protocol. TMA7 was labeled with the Pierce™ RNA 3' End Desthiobiotinylation Kit (Pierce, USA). The LSCC lysate was incubated with the purified biotinylated mixture at 4 °C for 1 h. Then, streptavidin-agarose beads were added to the lysate to precipitate the RNA-protein complexes. Then, the beads were washed and boiled in sodium dodecyl sulfate (SDS) buffer.

### Coimmunoprecipitation (Co-IP) assays

LSCC was lysed on ice for 30 min, and the supernatant was collected by centrifugation of the lysate at 14,000 ×g at 4℃ to estimate the protein concentration. Then, protein A+G magnetic beads were washed, resuspended with a TMA7 antibody, and incubated at 24 ℃ for 1 h. The supernatant was discarded by centrifugation, added the protein samples and the mixture was incubated on a shaker at 4 ºC overnight. Finally, the pellet was washed and boiled with SDS at 95 ºC for 5 min. The supernatant was analyzed by Western blotting.

### *In vivo* experiment

All animal experiments were performed in accordance with protocols approved by the Animal Care and Use Committee of Harbin Medical University. Male BALB/c nude mice (4-weeks-old; body weight 20-25 g; n=15) were obtained from Vital River Laboratories (Beijing, China) and maintained at 20-26 °C and 40-70% humidity with specific pathogen-free conditions. The mice were divided into control, TMA7 KD, and TMA7 KD+UBA2 OE groups. Each nude mouse was injected with 100 μL (1.0x10^7^ cells) of lentivirus-transfected LSCC cells. The formula length×width^ 2^×0.5 was used to calculate the tumor volume. 34 days after inoculation, the xenografts were excised, and their volume was determined.

### Transmission electron microscope (TEM)

LSCC cells were washed with PBS; a cell scraper was used to scrape the LSCC cells that were then collected by centrifugation at 1000 ×g at 4 ℃ for 15 min. The cells were fixed in 2.5% glutaraldehyde and 4 ℃ overnight, rinsed with 0.1 M PB at 4 ℃, 10-15 min, fixed with 2% osmic acid at room temperature for 1.5 h, washed, sectioned, stained with 2% uranyl acetate-lead citrate double staining, and images captured under a TEM.

### Statistical analysis

All statistical analyses were performed using GraphPad Prism version 8.0 and SPSS Statistics 17. Student's t-test was used to compare the two groups. Kaplan-Meier curves were used for survival analysis, and the log-rank test was used to compute the statistical significance. All quantitative experimental data were obtained from at least three replicates and expressed as mean±standard deviation. *p*<0.05 indicated significant significance (*p<0.05; **p<0.01; ***p<0.001).

## Supplementary Material

Supplementary figures 1, 3-6, 9, supplementary table 2.

Supplementary additional file 1, protein table.

Supplementary additional file 1, transcript table.

Supplementary additional file 2, table S2.

## Figures and Tables

**Table 1:** Relationship between TMA7 expression level and clinicopathological parameters of LSCC.

Characteristics (n)	TMA7 Expression		
	High	Low	*p*
Sex			0.249
Male (88)	41	47	
Female (27)	16	11	
Age			0.746
≥60 (75)	38	37	
<60 (40)	19	21	
T Classification			0.002**
T1-2 (59)	21	38	
T3-4 (56)	36	20	
Recrudescence			0.008**
Negative (78)	32	46	
Positive (37)	25	12	
Lymph node metastasis			<0.001***
Negative (68)	24	44	
Positive (47)	33	14	
Primary location			0.948
Supraglottic (38)	19	19	
Glottic (77)	38	39	
Clinical stage			<0.001***
I-II (62)	21	41	
III-IV (53)	36	17	

Patients with high TMA7 expression of LSCC could achieve a high clinical stage (***p<0.001) and T classification (**p<0.01). In addition, patients with high TMA7 had lymph node metastasis (***p<0.001) and frequent recurrence (**p<0.01).

## Abbreviations

TMA7: translation machinery associated 7 homolog
UBA2: ubiquitin like modifier activating enzyme 2
CCK-8: Cell Counting Kit-8
EdU: 5-Ethynyl-2′-deoxyuridine
DDP: Cisplatin
CRC: Colorectal Carcinoma
DMEM: Dulbecco's modified Eagle's medium
FBS: Fetal bovine serum
GO: Gene Ontology
KEGG: Kyoto Encyclopedia of Genes and Genomes
OD: Optical density
RIPA: Radioimmunoprecipitation assay
RNase R: Ribonuclease R
FTO: FTO alpha-ketoglutarate dependent dioxygenase
GAPDH: Glyceraldehyde-3-phosphate dehydrogenase
HNRNP: Heterogeneous nuclear ribonucleoprotein
HNSC: Head and neck squamous cell carcinoma
IGF2BP3: Insulin like growth factor 2 mRNA binding protein 3
IHC: Immunohistochemical
LSCC: Laryngeal squamous cell carcinoma
MeRIP: Methylated RNA immunoprecipitation
METT L14: Methyltransferase like 14
METTL16: Methyltransferase like 16
METT L3: Methyltransferase like 3
m6A: N6-methyladenosine
mRNA-seq: mRNA transcriptomic sequencing
OS: Overall Survival
PBS: Phosphate buffered saline
IHC: Immunohistochemical
RT-qPCR: real time quantitative polymerase chain reaction
RBM15: RNA binding motif protein 15
RFS: Relapse Free Survival
RIP: RNA immunoprecipitation
SCC: Squamous cell carcinomas
TMBIM6: Transmembrane BAX inhibitor motif containing 6
Wnt: Protein Wnt-2
WTAP: WT1 associated protein
YTHD F1: YTHN6-methyladenosine RNA binding protein 1
