# Supplementary material for: IGF2BP3 Regulates TMA7-mediated Autophagy and Cisplatin Resistance in Laryngeal Cancer via m6A RNA Methylation
Source: Int J Biol Sci. 2023 Feb 22;19(5):1382–400. doi: 10.7150/ijbs.80921 (PMC10086756; doi:10.7150/ijbs.80921)
Supplement: Supplementary file 1 — Supplementary figures 1, 3-6, 9, supplementary table 2. [file ijbsv19p1382s1.pdf]

Figure S1

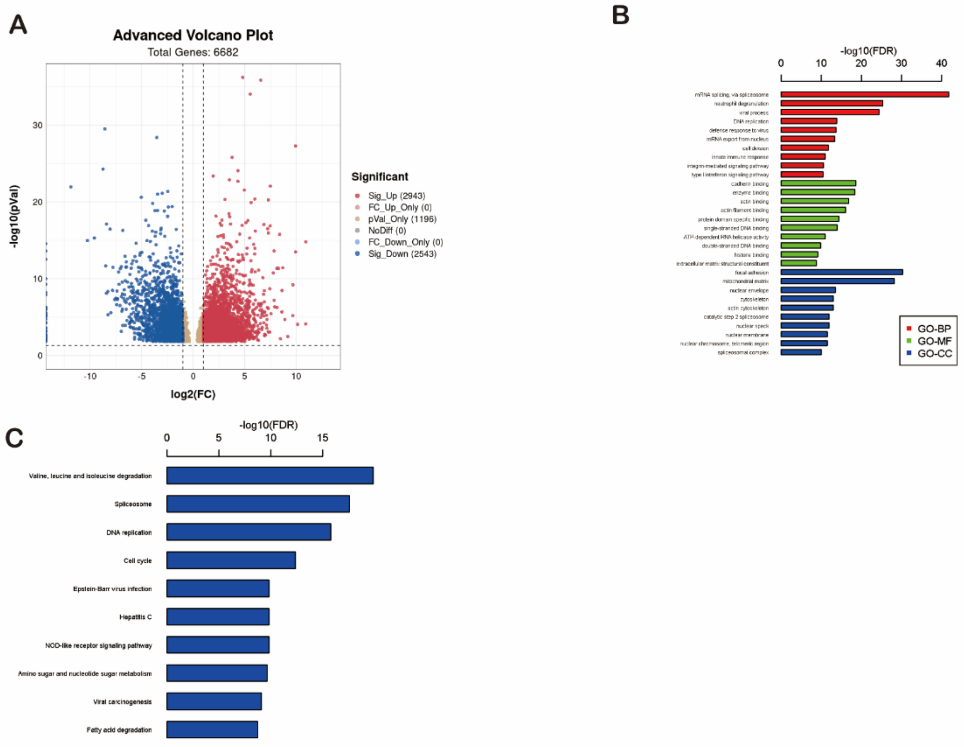

Fig. S1 a Transcriptomic volcano plot of differentially expressed genes. **b** and **c** Kegg analysis and go analysis of differentially expressed genes in LSCC identified by multi-omics analysis.

Figure S3

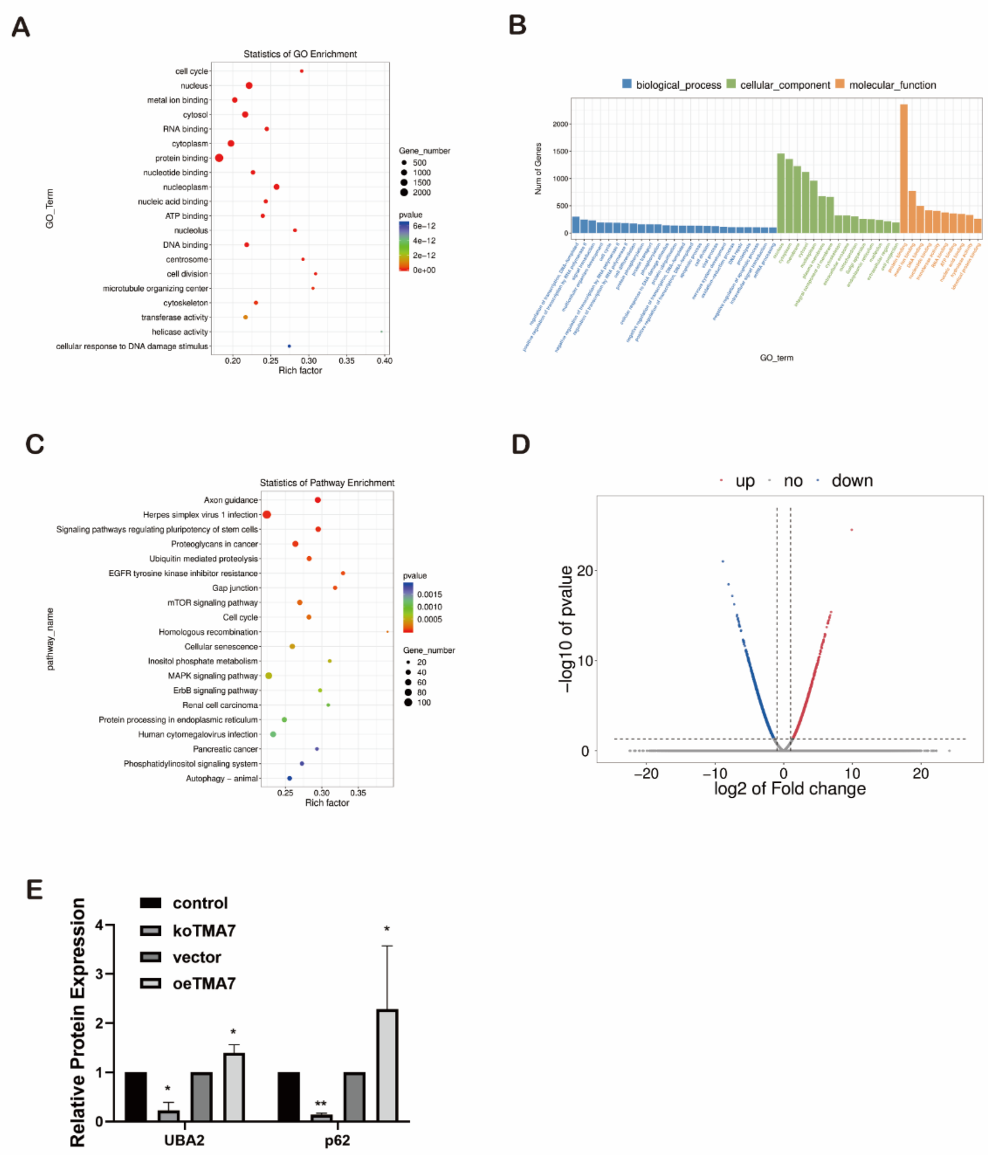

Figure S3 a and **b** Go analysis of differentially expressed genes in LSCC identified by multi-omics analysis. **c** Kegg analysis of differentially expressed genes in LSCC identified by multi-omics analysis. **d** Transcriptomic volcano plot of differentially expressed genes. **e** Relative protein expression after TMA7-KD or TMA7-OE.

Figure S4

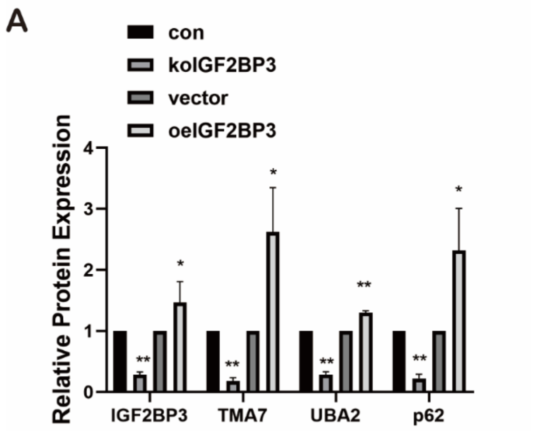

Fig. S4 a Relative protein expression after IGF2BP3-KD or IGF2BP3-OE.

Figure S5

A

#	Position	Sequence context	Structural context	Local structure visualization	Score(binary)	Score(knn)	Score(spectrum)	Score(combined)	Decision
1	80	UAAGU GUUCC GGAAC<br>CGUGA GGACU GCGGG<br>GACGG CGGGG UGGGG	PPBPP IPPPP PHHHH<br>PPBPP PIPPP PPMPP<br>PPBPP PPPPP PPPPM	Draw	0.707	0.774	0.543	0.644	m^6A site (**High** confidence)
2	329	AACAA AAAGA GGAGC<br>AGAAG AAACU CGAGG<br>AGCUA AAAGC GAAGG	PBPPP PPIII PPPPP<br>PPPPP PPMPP PPPPP<br>BPPPI IIIPP PHHHH	Draw	0.592	0.594	0.500	0.555	m^6A site (**Low** confidence)
3	465	AUGUC UUUUU CCUGC<br>CUCCU GAACU GCGAG<br>GUCUC GUUUU CCGAC	IPPPP PMPPP PPIPP<br>BPPPP PMMMM PPPPH<br>HHHPP PPMMP PPIPP	Draw	0.613	0.646	0.510	0.573	m^6A site (**Moderate** confidence)
4	2622	AACAU GGCAG UUAAG<br>AAUGU GGACU UUGAA<br>GUCAG AUGAC UCAGG	BBBBB PPPII PPPPP<br>PPIPP PPPMM MMPPP<br>PPPMM PPPII IPPPP	Draw	0.717	0.548	0.320	0.550	m^6A site (**Low** confidence)
5	3297	CUCAG AAAAA AAAAA<br>AAAAA GGACU UCUUU<br>UUCUG UAAAA UGGGU	MMMMM MMMMM MMMMM<br>MMMMM PPPPP PMMMM<br>MPPPP PIIII PPPPP	Draw	0.700	0.753	0.604	0.664	m^6A site (**High** confidence)

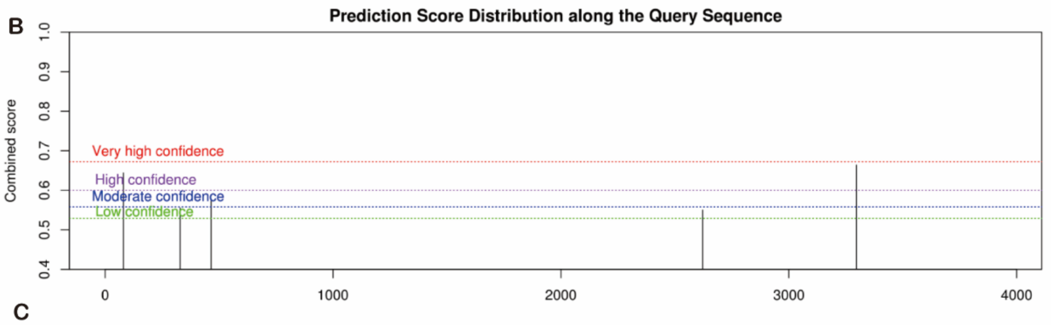

C

Log Likelihood Ratio: 64 **Information Content:** 30.4 **Relative Entropy:** 31 **Bayes Threshold:** 10.014

Name	Start	*p*-value	Sites
1. NC_000003.12:48440257-48444208	347	6.84e-11	AGCUAAAAGC GAAGGCCGCGGGGAAGGGGC CCUUGGGUAA
1. NC_000003.12:48440257-48444208	76	9.50e-11	CCGGAACCGU GAGGACUGCGGGGACGGCGG GGUGGGGACC
1. NC_000003.12:48440257-48444208	193	3.47e-10	AGGCCAAGGA GAUGGACGAGGUGAGGGCGG GCGCGGAGGC

Fig. S5 a, **b** and **c** Bioinformatic prediction of m6A modification in 3′-UTR of TMA7 mRNA.

Figure S6

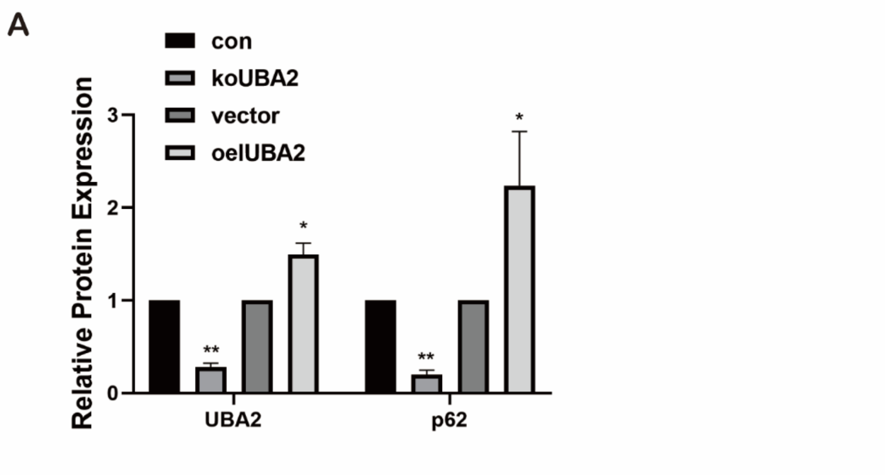

Fig. S6 Relative protein expression after UBA2-KD or UBA2-OE.

Figure S9

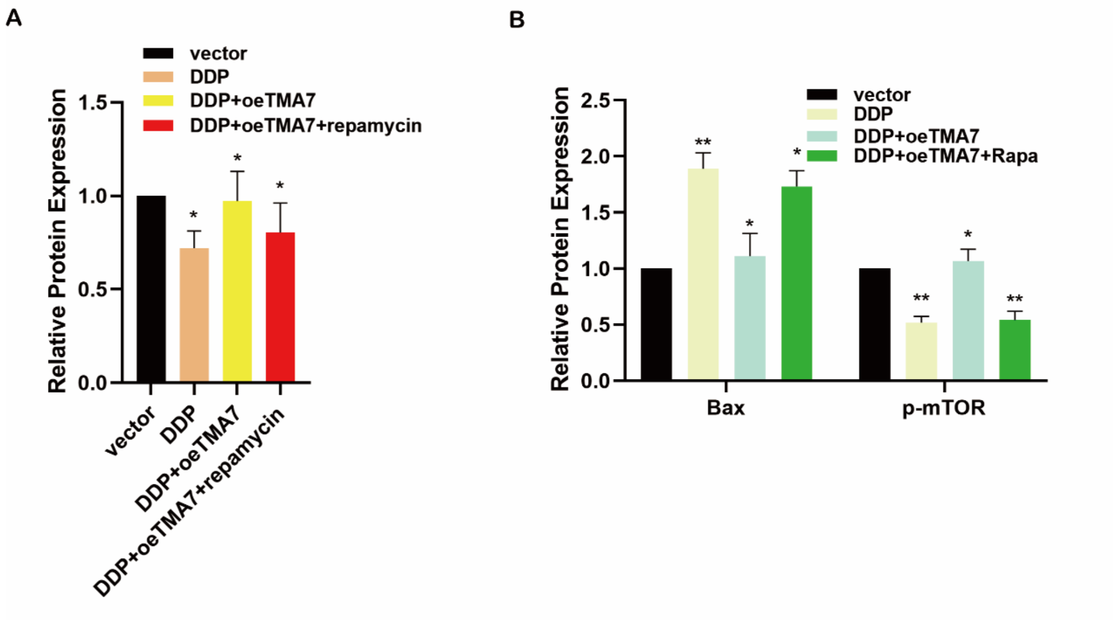

Fig. S9 Relative protein expression after the cells were treated with DDP TMA7-OE, rapamycin.

Supplementary Table 2 Primers used for RT-qPCR

Primers used for RT-qPCR

GAPDH F	5'-CGGATTTGGTCGTATTGGG-3'
GAPDH R	5'-CCTGGAAGATGGTGATGGG-3'
IGF2BP3 F	5'-GCACTTCCCTTTGTTGTAGTC-3'
IGF2BP3 R	5'-AGCACTTCCCTTAGGTTACTC-3'
TMA7 F	5'-GAAGGCCGCGGGGAAG-3'
TMA7 R	5'-GTCACCATCTCCTCAGGCAC-3'
UBA2 F	5'–CACAGGTTGCCAAGGAA–3'
UBA2 R	5'–GACACTCATAACACTCGGTCA–3'

Supplementary Table 2 These are the primers used for RT-qPCR
